# 39.3 CAN NEUROVIRAL INFECTIONS WITH HERPES SIMPLEX VIRUS, TYPE 1 (HSV-1) CONTRIBUTE TO RDOC?

**DOI:** 10.1093/schbul/sby014.161

**Published:** 2018-04-01

**Authors:** Vishwajit Nimgaonkar, Smita Deshpande, Triptish Bhatia, Konasale Prasad, Faith Dickerson, Raquel Gur, Ruben Gur, Kehui Chen, Joel Wood, Robert H Yolken

**Affiliations:** 1 University of Pittsburgh; 2 Dr. R.M.L. Hospital; 3 Indo-US Schizophrenia Study; 4 Sheppard Pratt; 5 University of Pennsylvania; 6 Johns Hopkins School of Medicine

## Abstract

**Background:**

The ‘viral hypothesis of schizophrenia’ is difficult to prove, partly because it is inherently difficult to find causes for chronic conditions. It is also not easy to date and relate the timing of infections or psychiatric diagnoses. Further, many neuroviruses cannot be isolated from infected persons. Progress could be made using RDoC for cognition as outcomes of infection, since those quantifiable variables cross psychiatric diagnostic domains (as do infections); they can also be monitored longitudinally and assessed with regard to treatment. We have tested this paradigm in relation to Herpes simplex virus, type 1 (HSV-1) that infects over 3.4 billion people. Though it can cause encephalitis, it is asymptomatic in the vast majority, only causing lifelong latent infection in neurons. Over 10 cross-sectional studies and longitudinal studies indicate that even in the absence of overt encephalitis, individuals seropositive for HSV-1 perform significantly worse than seronegative individuals in several cognitive domains, particularly those relating to memory. These associations remain significant following adjustment for demographic variables, such as socio-economic status. Even though post-mortem studies have been inconclusive, brain imaging studies also indicate gray matter volume reductions in frontotemporal regions in infected individuals, consistent with the cognitive impairment. Many neuronal models of latency are also available.

**Methods:**

In a prospective naturalistic follow up sample (PNFU), temporal changes in cognitive functions were analyzed in relation to baseline HSV-1 infection in persons with or without schizophrenia (N=226). Separately, in a randomized controlled trial (RCT), HSV-1 infected, clinically stabilized outpatients with SZ received Valacyclovir (VAL, an antiviral, 1.5 G twice daily for 16 weeks) or placebo (PLA) added to standard antipsychotic treatment, using a stratified randomization design, following placebo run-in (N=67). In both samples, HSV-1 infection (seropositivity) was estimated using serum IgG antibodies. All clinical evaluations were blinded to HSV-1 or treatment. Standardized Z scores for accuracy on eight cognitive domains were analyzed for temporal trajectories using generalized linear models (PNFU) and VAL/PLA differences compared with intent to treat analyses (RCT).

**Results:**

PNFU: At baseline, HSV-1 infected participants had significantly lower accuracy scores for Emotion Identification and Discrimination (EMOD), Spatial memory and Spatial ability (p=0.025, 0.029, 0.046, respectively), regardless of SZ diagnosis. They also had a significantly steeper temporal worsening for EMOD (p=0.03). RCT: EMOD improved significantly in VAL-treated patients (p=0.048, Cohen’s d=0.43).

**Discussion:**

HSV-1 infection is associated with time-related dysfunction in EMOD, which indexes social cognition. Conversely, VAL treatment improves EMOD. A portion of HSV-1 associated cognitive dysfunction is progressive, but remediable. Viral infections could be used to investigate and validate RDoC criteria.

